# A Novel AlGaN/Si_3_N_4_ Compound Buffer Layer HEMT with Improved Breakdown Performances

**DOI:** 10.3390/mi13030464

**Published:** 2022-03-18

**Authors:** Jingwei Guo, Shengdong Hu, Ping Li, Jie Jiang, Ruoyu Wang, Yuan Wang, Hao Wu

**Affiliations:** 1 Chongqing Engineering Laboratory of High Performance Integrated Circuits, School of Microelectronics and Communication Engineering, Chongqing University, Chongqing 400044, China; cqu_gjw@163.com (J.G.); lipingstu@cqu.edu.cn (P.L.); jiangjie95518@163.com (J.J.); 20163874@cqu.edu.cn (R.W.); wangyuan0320@gmail.com (Y.W.); wuhao_shane@163.com (H.W.); 2 China Resources Microelectronics (Chongqing) Limited, Chongqing 401332, China

**Keywords:** AlGaN/GaN HEMT, breakdown voltage, buffer layer, electric field

## Abstract

In this article, an AlGaN and Si_3_N_4_ compound buffer layer high electron mobility transistor (HEMT) is proposed and analyzed through TCAD simulations. In the proposed HEMT, the Si_3_N_4_ insulating layer is partially buried between the AlGaN buffer layer and AlN nucleating layer, which introduces a high electric field from the vertical field plate into the internal buffer region of the device. The compound buffer layer can significantly increase the breakdown performance without sacrificing any dynamic characteristics and increasing the difficulty in the fabrication process. The significant structural parameters are optimized and analyzed. The simulation results reveal that the proposed HEMT with a 6 μm gate-drain distance shows an OFF-state breakdown voltage (BV) of 881 V and a specific ON-state resistance (R_on,sp_) of 3.27 mΩ·cm^2^. When compared with the conventional field plate HEMT and drain connected field plate HEMT, the breakdown voltage could be increased by 148% and 94%, respectively.

## 1. Introduction

Gallium nitride (GaN) devices are widely used in the high-frequency and high-power fields of power electronics due to their superior material properties, such as a large band gap, high critical electric field, good thermal conductivity, and high electron saturation speed [1,2,3,4,5]. AlGaN/GaN high electron mobility transistor (HEMT) devices are fabricated by growing a thin AlGaN barrier layer on the GaN channel layer based on a transition layer [6,7]. The strong polarization effect between AlGaN and GaN will confine the electrons at the surface of the GaN channel, thereby forming a 2-dimensional electron gas (2DEG) with high mobility [8,9]. At the same time, GaN-on-silicon is widely used owing to its low cost and large size, which can be integrated with Si-CMOS technology [10,11]. In recent years, there have been an increasing number of reports on high-performance AlGaN/GaN HEMT devices [12,13,14,15,16,17], however, there is still a large gap between the limits of GaN material properties and commercial devices. Researchers mainly use field plate technology or progressive transition layers to obtain a low specific on-resistance (R_on,sp_) with a high breakdown voltage (BV), thereby realizing a higher figure-of-merits (FOM) (FOM = BV^2^/R_on,sp_). The field plate is capable of alleviating the electrical field crowding at the drain side of the gate edge. At the same time, the field plate introduces a new electrical field peak at its edge, which has been proven to significantly improve the BV [18,19,20]. In the fabrication process, the field plate is connected to the gate and placed over the passivation layer. However, the improvement of the device performance depends on the lateral length of the field plate, and the breakdown voltage rises at the beginning and then declines with increasing field plate [11,12,13,14,15,16,17,18,19,20,21]. At the same time, the field plate is equivalent to increasing the electrode overlapping area, which brings the corresponding parasitic capacitance and deteriorates the switching performance of the device. Recently, an AlGaN or GaN buffer layer is substituted by an AlN back barrier layer that has been fabricated and demonstrated to have a high BV and FOM [22,23,24]. Utilizing the characteristics of AlN with an ultra-large bandgap energy (6.2 eV) and polarization effect, the confinement effect of 2DEG is increased. Therefore, a higher BV and a lower R_on,sp_ can be obtained. However, GaN HEMTs with an AlN back barrier architecture will introduce a negative bound charge between the GaN and AlN, which will deplete the channel 2DEG, reduce the channel electron density, and exacerbate the output performance [25]. On the other hand, the interface of the AlN and silicon substrate will form the inversion electron layer in the buffer region, which will increase the leakage of the device and block the expansion of the depletion layer, thus affecting the breakdown characteristics [26]. To eliminate these adverse effects, the AlN layer needs to be meticulously designed and optimized, which will undoubtedly increase the cost and process complexity.

Recently, a novel drain-connected field plate GaN HEMT (DC-HEMT) was proposed, which improved the breakdown and radio frequency power amplifier performance by taking advantage of a vertical-field plate [21]. However, the withstand voltage in the buffer region remains limited. Therefore, this paper proposes a novel high-performance HEMT with a compound buffer layer, which changes the conventional AlGaN or GaN buffer layer into an AlGaN and Si_3_N_4_ compound buffer layer. On the basis of adopting the vertical drain field plate structure, a high electric field on the device surface is introduced into the buffer region. At the same time, the compound buffer layer increases the insulation and reduces the buffer leakage. In this paper, the internal mechanism of the device structure is comprehensively analyzed by numerical simulation, and the parameters of the device are optimized. Finally, the optimized proposed HEMT obtains a BV of 881 V and a R_on,sp_ of 3.27 mΩ·cm^2^ at a gate drain distance (L_GD_) of 6 μm. This paper is arranged as follows. The second chapter introduces the structure and mechanism analysis of the proposed method. Then, the third chapter presents the simulation results and optimizes the important parameters. Finally, the last chapter provides a conclusion.

## 2. Device Structure and Mechanism

**Figure 1:**
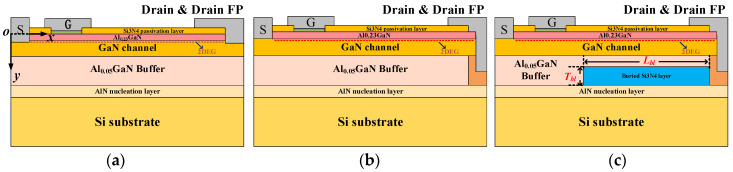
Schematic cross-section of the (**a**) Con-HEMT, (**b**) DC-HEMT, and (**c**) proposed buried Si_3_N_4_ passivation layer GaN HEMT (BP-HEMT).

The schematic cross-section of the proposed buried Si_3_N_4_ passivation layer GaN HEMT (BP-HEMT) is shown in Figure 1c. For comparison, the conventional drain field plate HEMT (Con-HEMT) and DC-HEMT are given in Figure 1a,b, respectively. One available fabricating process flow of the proposed GaN HEMT is introduced as summarized below. Firstly, A 50 nm AlN nucleation layer is grown by metal-organic chemical vapor deposition (MOCVD) on a 3 μm n-type silicon substrate. Secondly, the buried Si_3_N_4_ layer is grown at high temperature in the same MOCVD chamber with silane and ammonia. Then, the Si_3_N_4_ layer under the source side is removed selectively by using reactive ion etching (RIE) and inductively coupled plasma (ICP) etch. After that, the low-k benzocyclobutene (BCB) planarization is used to avoid the roughness between the Si_3_N_4_ and AlGaN. Finally, the epilayers and AlGaN/GaN heterojunction are regrown as the common GaN HEMTs [27,28,29]. All studied HEMTs consist of a 0.2-μm passivation layer on a 15 nm Al_0.23_GaN barrier layer, a 200-nm GaN channel, a 2 μm silicon-doped Al_0.05_GaN buffer layer.

**Figure 2:**
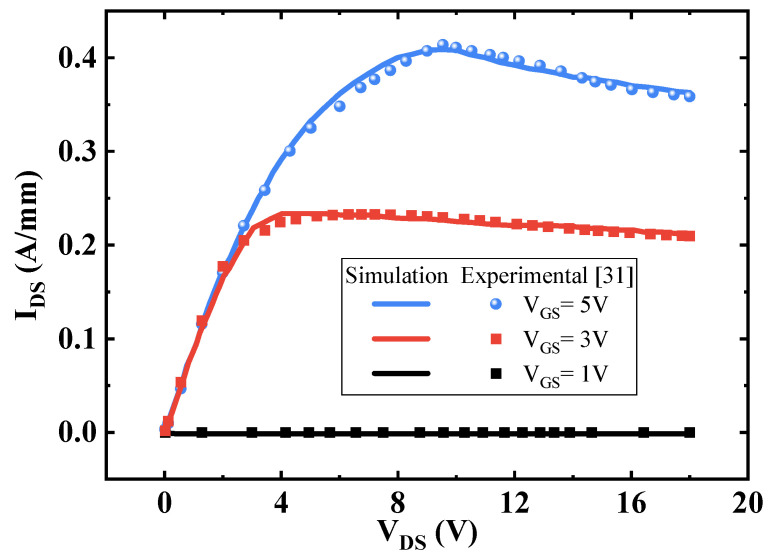
Experimental [31] and numerical simulation on-state characteristic curves at V_GS_ = 1, 3, 5 V.

In this study, the internal mechanism of HEMTs is simulated and analyzed by TCAD Sentaurus software from Synopsys Inc. (Mountain View, CA, USA) [30]. The drain and source electrodes are set to ohmic contacts. For the P-type GaN gate electrode, it is set to a Schottky contact. A thermal contact is set for the bulk electrode under the substrate. The necessary physical models are considered in the simulation, including the piezoelectric polarization model, anisotropy of materials, Shockley–Read–Hall (SRH) recombination model, avalanche model, mobility model considering doping-dependent degradation and high electric field velocity saturation, carrier tunneling model, and no band gap narrowing model. The electron states of the 2DEG are computed by using the model of spontaneous and piezoelectric. The 2DEG density is calculated based on the AlGaN barrier mole fraction and the strain resulting from the lattice constant. The van Dort model is as the quantization model in simulations because it is a numerically robust, fast, and proven model. The electron–electron interaction in the 2DEG is considered in the recombination and mobility models. The significant incomplete ionization of Mg ions in GaN is also considered. Figure 2 compares the experimental results from Oliver Hilt [31] and simulation results when the device is on state at V_GS_ = 1, 3, 5 V and L_GD_ = 6 μm. These models added in the simulations are adopted according to [32,33]. As shown in Figure 2, the I_DS_-V_DS_ simulation characteristics fit well with experimental results. The R_on,sp_ also evidently indicates a good agreement between simulated and experimental data. A fixed acceptor trap concentration of 1 × 10^18^ cm^−3^ and a fixed donor trap concentration of 1 × 10^13^ cm^−2^ between the AlGaN barrier layer and passivation layer are equipped simultaneously [34,35]. The lateral dimensions and other doping characteristics of the devices are given in Table 1.

**Figure 3:**
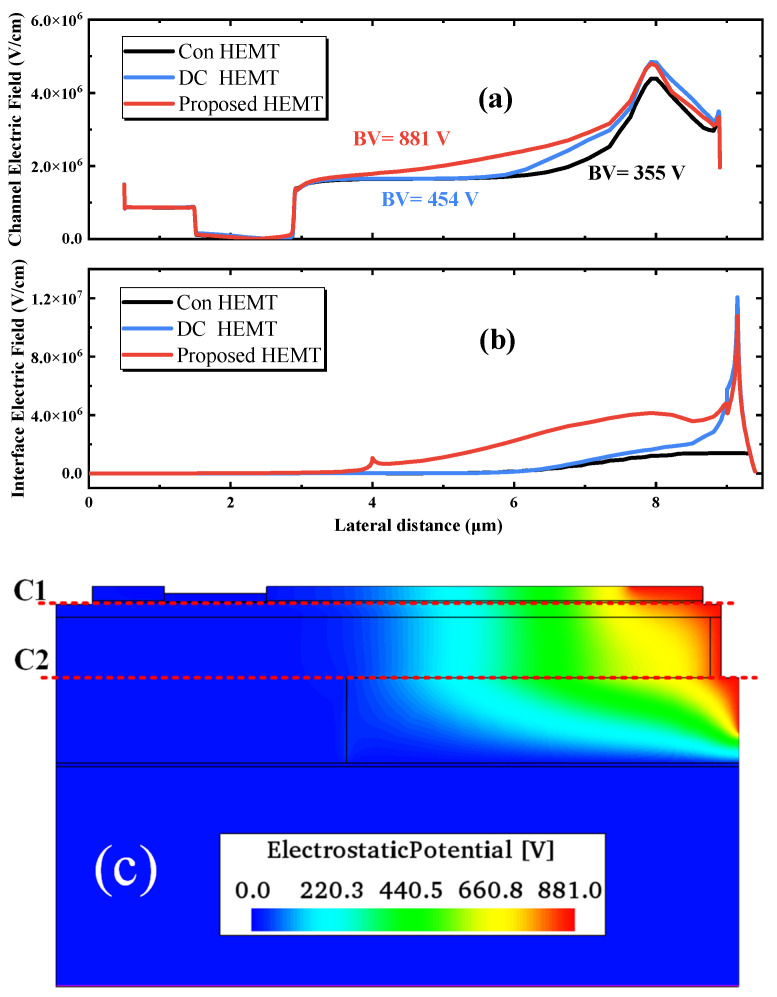
Simulated lateral electric field profile at the (**a**) channel and the (**b**) interface of the AlGaN buffer layer and buried Si_3_N_4_. (**c**) The specific cutlines along the device and the electrostatic potential distribution of the proposed HEMT.

**Figure 4:**
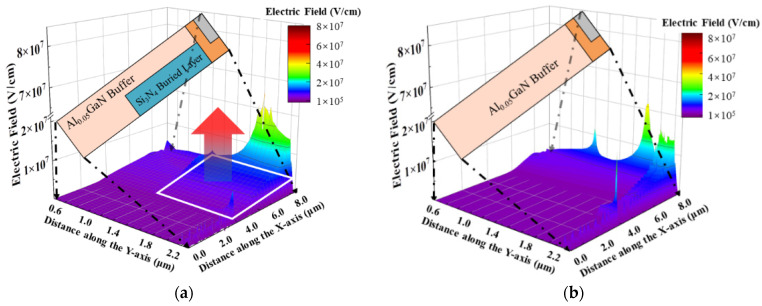
The 2-D electric field distribution in the whole buffer region of (**a**) BP-HEMT and (**b**) DC-HEMT.

Figure 3 compares the lateral electric field profile at the (a) channel, cutline C1 in Figure 3c and (b) the interface between the AlGaN buffer layer and Si_3_N_4_ buried layer, cutline C2 in Figure 3c when the devices have avalanche-induced breakdown in the off state (the criteria are set when the drain current reaches 0.01 mA/mm). The electrostatic potential distribution of the proposed HEMT is also shown in Figure 3c. Comparing Figure 3a,b, it can be seen that the maximum electric field peak of the Con-HEMT is located at the edge of the drain field plate, and the electric field in the buffer region is smaller. For DC and BP HEMTs, they have not only the lateral field plates but also vertical field plates. The vertical field plate transports the high electric field into the buffer region, with the additional high electric field far away from the channel assisting in suppressing the impact ionization. Consequently, the crowded electric field at the drain electrode and field plate is alleviated, and the breakdown voltage is enlarged. In BP-HEMT, the inserted Si_3_N_4_ layer could sustain a larger electric field due to its larger band gap. It takes along the high electric field at the vertical field plate into the interior of the buffer region and therefore further enhances the electric field at the channel and the interface contrast with the DC-HEMT, as shown in Figure 3a,b. Figure 4 demonstrates the 2-D electric field distribution in the whole buffer region of (a) BP-HEMT and (b) DC-HEMT, and the electric field corresponding to the Si_3_N_4_ buried layer in the buffer region of BP-HEMT is greater than that in the single AlGaN buffer region of DC-HEMT. In summary, the novel proposed BP-HEMT employs a Si_3_N_4_ buried layer to further introduce a high electric field into the buffer region, which improves the breakdown performance without sacrificing the on-state performance.

## 3. Simulation Results and Discussion

**Figure 5:**
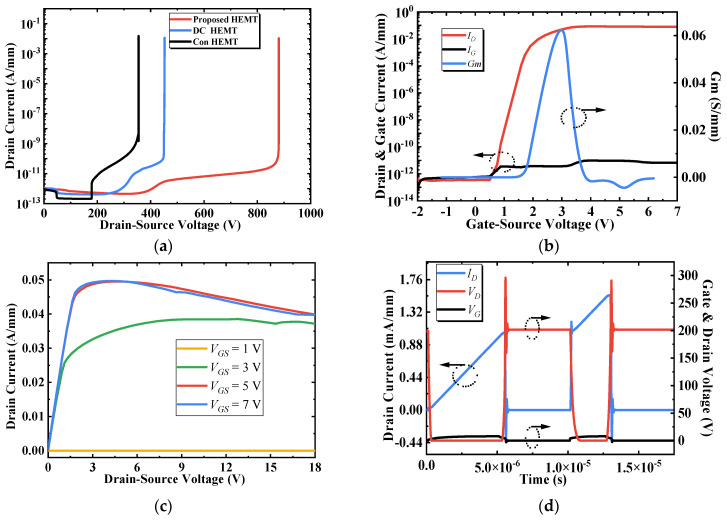
Electrical characteristics of devices. (**a**) Breakdown performance of Con, DC, and proposed BP-HEMTs. (**b**) Transfer and transconductance characteristics of BP-HEMT. (**c**) Output *I_D_*-*V_D_* characteristics of BP-HEMT. (**d**) Switching characteristics of BP-HEMT.

The breakdown characteristics of the devices are compared in Figure 5a. The breakdown voltages are 355, 454, and 881 V for Con, DC and the proposed BP-HEMTs, respectively. Compared with Con and DC-HEMTs, the BV of BP-HEMT is improved by 148% and 94%, respectively. The transfer and transconductance (defined as the calculated derivative of drain current with respect to gate-source voltage, d*I_DS_*/d*V_GS_*) of BP-HEMT are simulated at *V_DS_* = 15 V, and the gate voltage sweeps from −2 to 7 V. As shown in Figure 5b, the threshold voltage is 1.7 V, and the maximum transconductance is 0.062 S/mm. Figure 5c shows the on-state *I_D_*-*V_D_* characteristics of BP-HEMT with drain-to-source voltages that range from 0 to 18 V at *V_GS_* = 1, 3, 5, and 7 V. The current collapse phenomenon occurs when *V_GS_* > 3 V and occurs similarly in Con and DC HEMTs. The reason for the current collapse is that the electrons in the channel will be injected into the adjacent AlGaN barrier region and captured by deep traps as the *V_DS_* increases. The specific on-state resistance is 3.27 mΩ·cm^2^ for BP-HEMT and an approximate value for Con and DC HEMTs because the buried Si_3_N_4_ layer has no influence on the output current. Figure 5d shows the switching characteristics of the BP-HEMT when a double pulse is applied at a supply voltage of 200 V. The high-speed switching performance is preserved in the BP-HEMT.

**Figure 6:**
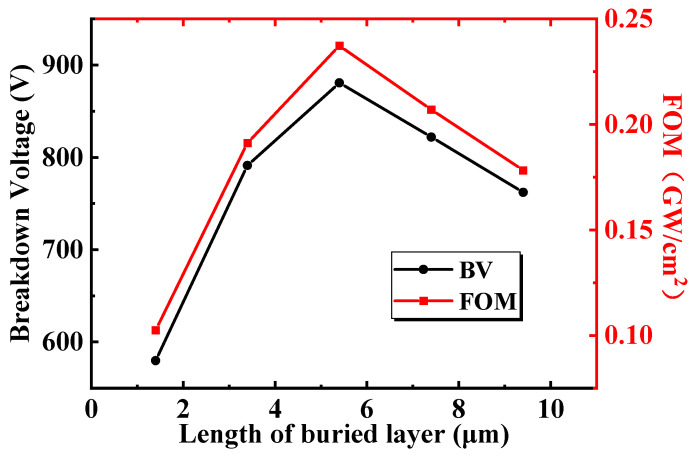
The influence of the length of the buried layer on the breakdown voltage and the FOM of the proposed BP HEMT.

**Figure 7:**
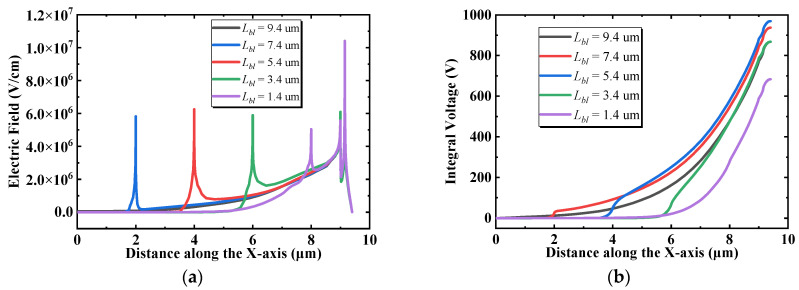
(**a**) The off-state lateral electric field distribution with different *L_bl_*. (**b**) The integral of the electric field along the *X*-axis with different *L_bl_*.

Figure 6 shows the influence of *L_bl_* (the length of the buried layer) on the BV and FOM of the BP-HEMT. The BV and FOM initially increase and then decrease while the *L_bl_* rises, and the maximum values are observed to be *L_bl_* = 5.4 μm. For further investigations, Figure 7a illustrates the off-state lateral electric field distribution at the interface between the Si_3_N_4_ buried layer and AlGaN buffer layer under a drain voltage of 500 V with different *L_bl_*. In every single profile, the left electric field of the peak is sustained by the AlGaN buffer region, and the right electric field is maintained by Si_3_N_4_. It is apparent that the electric field in Si_3_N_4_ is higher than that in AlGaN. On the other hand, in the interior of the Si_3_N_4_ layer, the magnitude of the electric field declines progressively from the vertical field plate to the center of the buffer region. Therefore, when Si_3_N_4_ is shorter, the length of the high electric field is extremely small, and when Si_3_N_4_ is longer, nevertheless, the average electric field in the Si_3_N_4_ buried layer has a declining trend. Figure 7b calculates the integration of the electric field of Figure 7a, and the integral voltage means the magnitude of the supposed voltage in this interface. The maximum integral voltage is obtained when *L_bl_* = 5.4 μm, and the minimum value is attained when *L_bl_* = 1.4 μm. Moreover, the calculated integral voltages in Figure 7b that correspond to *L_bl_* are in perfect agreement with the BV, as shown in Figure 6. In summary, *L_bl_* has a significant effect on the average electric field. Furthermore, the supposed voltage in the buffer layer is altered. However, it should be noted that the maximum integral voltage is not equal to the drain bias voltage of the device because the total voltage is supported by lateral and vertical electric field and it is only used to compare the magnitude of the voltage assumed in the interface with different *L_bl_*. In the on state, because the Si_3_N_4_ buried layer hardly affects the concentration and mobility of 2DEG, R_on,sp_ is not determined by *L_bl_* accordingly. *L_bl_* only slightly affects the magnitude of the saturation output current, which can be neglected.

**Figure 8:**
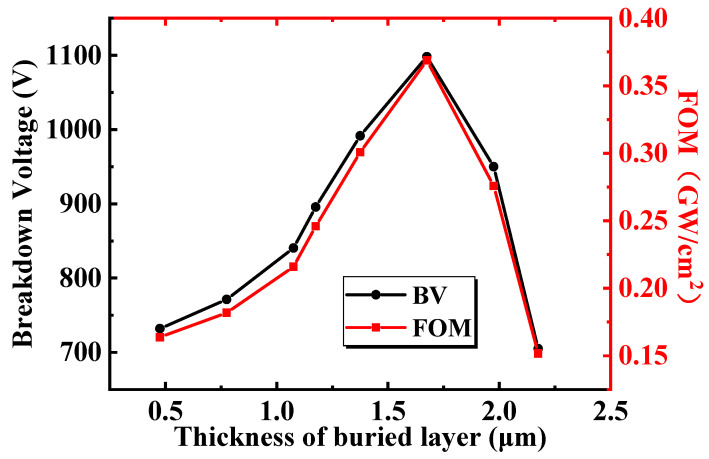
The influence of the thickness of the buried layer on the breakdown voltage and the FOM of the proposed BP HEMT.

Figure 8 shows the variation in *T_bl_* (the thickness of the buried layer) on the BV and FOM of the proposed BP-HEMT. Apparently, BV and FOM first increased and then decreased as *T_bl_* increased. When *T_bl_* reaches 1.675 μm, BV and FOM reach maxima of 1098 V and 0.37 GW/cm^2^, respectively. Additionally, as mentioned previously, when analyzing *L_bl_*, R_on,sp_ is almost independent of the Si_3_N_4_ buried layer. Therefore, the following analyses concentrate on the BV as influenced by *T_bl_*.

**Figure 9:**
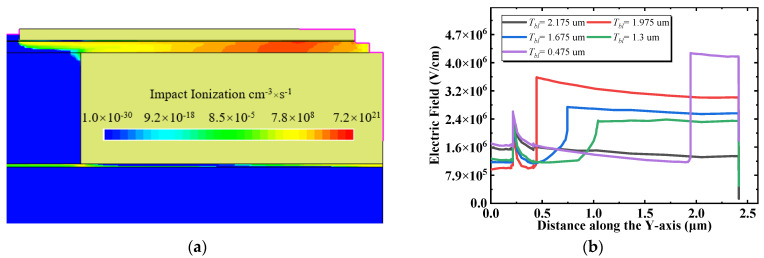
(**a**) Vertical electric field distribution versus distance along the *Y*-axis for various Si_3_N_4_ buried layer thicknesses. (**b**) Impact ionization rate with a buried layer thickness of 2.175 μm.

Figure 9a shows the vertical electric field distribution at the location of x = 7.9 μm in the off state with a drain voltage of 700 V, and *T_bl_* ranges from 0.475 to 2.175 μm. It should be noted that the region beneath the buffer layer does not sustain the reverse voltage. Therefore, the electric field in the substrate layer is not displayed. The electric field in the Si_3_N_4_ layer is higher than that in the AlGaN buffer region and an electric field peak is introduced at the interface between the AlGaN and Si_3_N_4_ buried layers. When the Si_3_N_4_ layer is thin (*T_bl_* = 0.475 μm), the electric field peak neighbors the vertical field plate and is farther from the horizontal field plate and the drain electrode, and thus, it has the highest electric field peak, but the width with a high electric field is the narrowest, which equals *T_bl_*. As the thickness of the Si_3_N_4_ layer increases, the distance from the high electric field produced by the vertical field plate is far removed. At the same time, the distance from the horizontal field plate and the drain electrode is adjacent, and the electric field peak consequently increases. The width of the high electric field also increases. Since the electric field peak at the horizontal field plate is smaller than the vertical field plate, the interface electric field peak caused by the horizontal field plate is also small. For that reason, the strength of the average electric field depends on both the width and height of the high electric field, and it rises first and reduces later, along with a similar trend in the BV and FOM. On the other hand, when the Si_3_N_4_ layer is sufficiently thick and close to the 2DEG, the high electric field will accelerate the carriers in the channel, promoting the occurrence of avalanche ionization and consequently reducing BV. Figure 9b demonstrates the impact ionization rate under a drain voltage of 700 V in the off state at *T_bl_* = 2.175 μm. The thicker Si_3_N_4_ layer confines the impact ionization in the channel, and it brings about a premature breakdown. The above two reasons cause the BV and FOM to decrease after an initial increase.

**Figure 10:**
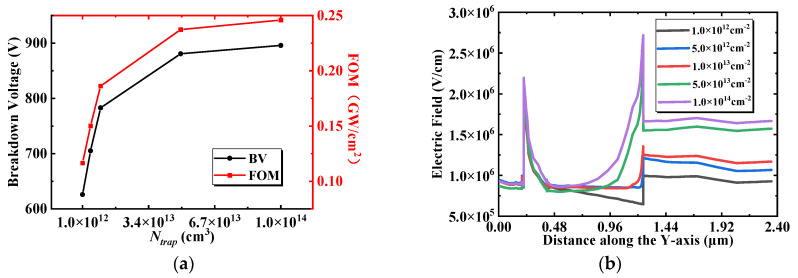
(**a**) Dependence of BV and FOM on the interface trap concentration of the AlGaN buffer region and Si_3_N_4_ buried layer. (**b**) Vertical electric field at x = 6.0 μm in the off state with a drain voltage of 600 V.

The SiN*_x_* passivation layer on the surface of the device is widely utilized to suppress the current collapse, and the interface states between the SiN*_x_* and AlGaN barrier layers have been broadly investigated [32,33,34,35]. The existence of donor-type traps between the interface has been demonstrated, and in the proposed BP-HEMT, donor-type traps are introduced correspondingly at the interface between the AlGaN buffer and Si_3_N_4_ buried layer during fabrication. To further analyze the effect of interfacial donor traps on the breakdown performance of the device, a fixed energy level interfacial donor trap is set between the SiN*_x_* and AlGaN simultaneously during the simulation. Figure 10a shows the dependence of BV and FOM on the interface trap concentration (*N_trap_*) of the AlGaN buffer region and Si_3_N_4_ buried layer. The BV and FOM increase rapidly and then stabilize as *N_trap_* increases. To analyze the reasons, Figure 10b shows the vertical electric field at x = 6.0 μm in the off state with a drain voltage of 600 V, and for the same reason as Figure 9a, the region below the nucleation layer is not plotted. The trap located at the interface of the AlGaN buffer and Si_3_N_4_ buried layer is farther away from the channel, without affecting the concentration of 2DEG, and it is quite distinct from the passivation layer trap. Therefore, the vertical electric field peak at the channel (the first peak in Figure 10b) is not affected by *N_trap_*. The electric field at the interface between the AlGaN buffer and the Si_3_N_4_ buried layer (the second peak in Figure 10b) and the magnitude of the electric field in the Si_3_N_4_ buried layer increase significantly with increasing *N_trap_*. The reason for this variation is that the high concentration donor leaves more positive fixed charges at the interface after ionizing the electrons, and more electric field lines pass through the Si_3_N_4_ buried layer to the interface from the edge of the DC field plate. With an increased electric field in the Si_3_N_4_ buried layer, the voltage sustaining capability of the whole device is increased significantly. The above analysis shows that the device performance does not deteriorate even if there is a higher *N_trap_* due to the lattice and thermal mismatch, and the proposed BP-HEMT shows superior process tolerances.

## 4. Conclusions

This paper presents a novel normally-off p-type AlGaN/GaN HEMT with a Si_3_N_4_ and AlGaN compound buffer layer. The proposed method can enhance the BV and FOM, which takes advantage of lattice matching in Si_3_N_4_ and AlGaN. The proposed BP-HEMT alleviates the contradiction that the high electric field caused by the drain-connected field plate would reduce the BV of the device and introduces a high electric field into the buffer region without sacrificing the on-state output current capability and switching performance. After optimizing the parameter, a BV of 881 V and FOM of 0.24 GW/cm^2^ are obtained. Simultaneously, the proposed BP-HEMT will not bring additional challenges to the device process, and it is expected to become a strong competitor of high-power GaN devices and circuits.

## Figures and Tables

**Table 1:** Parameters of Con, Dc, and Proposed BP-HEMT.

Parameters	Con-HEMT	DC-HEMT	BP-HEMT
Gate-to-source length, *L_GS_* (μm)	1	1	1
Gate-to-drain length, *L_GD_* (μm)	6	6	6
Length of p-type GaN gate (μm)	1.4	1.4	1.4
Length of drain field plate (μm)	1	1	1
Doping concentration of channel (cm^−3^)	1 × 10^15^	1 × 10^15^	1 × 10^15^
Doping concentration of buffer (cm^−3^)	1 × 10^14^	1 × 10^14^	1 × 10^14^
Doping concentration of substrate (cm^−3^)	1 × 10^15^	1 × 10^15^	1 × 10^15^
Width of vertical Si_3_N_4_ layer (μm)	-	0.15	0.15
Length of buried Si_3_N_4_ layer (μm)	-	-	5.4
Thickness of buried Si_3_N_4_ layer (μm)	-	-	1.675
